# *In “Vitro”* Lps-Stimulated Sertoli Cells Pre-Loaded With Microparticles: Intracellular Activation Pathways

**DOI:** 10.3389/fendo.2020.611932

**Published:** 2021-01-07

**Authors:** Iva Arato, Domenico Milardi, Stefano Giovagnoli, Giuseppe Grande, Catia Bellucci, Cinzia Lilli, Sara Bartoli, Sara Corneli, Piera Mazzone, Mario Calvitti, Tiziano Baroni, Riccardo Calafiore, Francesca Mancuso, Giovanni Luca

**Affiliations:** ^1^ Department of Experimental Medicine, University of Perugia, Perugia, Italy; ^2^ International Scientific Institute “Paul VI”, Rome, Italy; ^3^ Division of Endocrinology, Fondazione Policlinico Universitario “Agostino Gemelli”, Rome, Italy; ^4^ Department of Pharmaceutical Sciences, University of Perugia, Perugia, Italy; ^5^ 2nd Department of Medicine, “Ca’ Foncello” Regional Hospital, ULSS2 Marca Trevigiana, Treviso, Italy; ^6^ Istituto Zooprofilattico Sperimentale dell'Umbria e delle Marche "Togo Rosati", Perugia, Italy; ^7^ Department of Medicine, University of Perugia, Perugia, Italy; ^8^ Division of Medical Andrology and Endocrinology of Reproduction, Saint Mary Hospital, Terni, Italy

**Keywords:** Sertoli cells, lipopolysaccharide, microparticles, pro-inflammatory pathways, nonprofessional tolerogenic

## Abstract

Sertoli cells (SC) are immune privileged cells with the capacity of modulating the immune response by expressing several immune-regulatory factors. SC have the capacity to respond to external stimuli through innate phagocytic and antibacterial activities. This evidence evoked a potential role of SC as drug carriers and therapeutic agents. Such stimuli drive SC towards a still unknown evolution, the clinical relevance of which as yet remains undisclosed. This study sought to investigate the effects of external stimuli in the form of polymeric microparticles (MP) and bacteria derived endotoxins, such as lipopolysaccharides (LPS), in order to identify the pathways potentially involved in cell phenotype modifications. Compared to single stimulation, when combined, MP and LPS provoked a significant increase in the gene expression of IDO, PD-L1, FAS-L, TLR-3, TLR-4, MHC-II, ICAM-1, TFGβ1, BDF123, BDF129, BDF3 and pEP2C. Western Blotting analysis demonstrated up-regulation of the ERK 1–2 and NF-kB p65 phosphorylation ratios. Our study, showing the exponential increase of these mediators upon combined MP and LPS stimulation, suggests a “switch” of SC function from typical cells of the blood-testicular barrier to nonprofessional tolerogenic antigen-presenting cells. Further studies should target the clinical and technological implications of such stimuli-induced SC transformation.

## Introduction

Sertoli Cells (SC), located in the seminiferous epithelium, are somatic “nursing” cells that mechanically segregate germ cell autoantigens by means of the blood-testis barrier (BTB) and create a microenvironment that protects the germ cells from the host’s immune system attack on their development within the tubules (1, 2).

SC not only serve as a physical barrier, but can enable modulation of the immune response as well by secreting trophic, anti-inflammatory, and immunomodulatory factors (3). In particular, SC produce different hormones, including the anti-Müllerian hormone (AMH), Activin A, and inhibin B that play an important role for the preservation of their function (4).

Additionally, other cytokines and immunomodulatory factors, including the Transforming Growth Factor-β (TGFβ), Interleukin (IL)-1α and IL-6, defensins (α-, β-defensin), Indoleamine 2,3-dioxygenase (IDO), and the presence of several toll-like receptors contribute to SC immune competence (5–8). Due to these properties, SC have been used for different therapeutic applications, in allogeneic and xenogeneic transplant protocols as well as for the management of several chronic diseases (9–15).

The multifaceted SC nature enables them to respond to multiple stimuli and conditions. As a result, SC can accomplish a number of fundamental roles, including scavenger activities during spermatogenesis and infections.

These innate phagocytic and antibacterial aptitudes further make SC therapeutically attractive.

SC provide the first line of response to invading pathogens gaining access *via* the male genital tract (16, 17).

A bacteriostatic action of rat SC was postulated against phagocytized *Staphylococcus aureus* (18) and ascribed to the secretion of defensins in rodents and canines (19, 20) and to the high mobility group box chromosomal protein 1 in rat and human SC (21).

Moreover, SC have been found potentially useful as safe drug carriers against inflammatory conditions and infections. Kumar et al., transplanted rat SC pre-loaded with curcumin containing chitosan nanoparticles, in a mouse model of acute pulmonary inflammation.

SC accumulated preferentially in the lungs with no immune complications or other observed side effects (22). In another study, the SC phagocytic and antibacterial activities were exploited to formulate a SC-based drug delivery system (23). The SC loaded with a microencapsulated antibiotic complex showed good antibacterial activity over time and storage potential, posing the base for the development of novel cell-based drug delivery systems.

Nevertheless, even though most of SC functions were preserved, it is conceivable to suppose that such a manipulation may induce significant changes in cell characteristics. Moreover, whether such changes may occur even upon external stimulation by toxicants during infection or exposure to non-toxic contaminants in the testis is not known.

Being the whole picture rather unclear and, to the best of our knowledge, not been explored so far, this work was focused on the understanding of how external stimuli may alter SC features and induce SC evolution towards a mutated phenotype, which may suggest potential clinically relevant health consequences.

On this purpose, lipopolysaccharides (LPS), potent pro-inflammatory bacterial endotoxins from the cell wall of Gram-negative bacteria, were chosen as an infection-related stimulus, since they are generally recognized as a standard model for investigating inflammation and response to infection *in vivo* and *in vitro* (24, 25). In fact, LPS can initiate a strong immune response and serve as an early warning signal of bacterial infection. Circulating LPS are intercepted by the LPS binding protein (LBP) in the serum (26), which then transfers LPS to CD14 that splits LPS aggregates into monomeric molecules and presents them to the TLR4–MD-2 complex. Aggregation of the TLR4–MD-2 complex after LPS binding leads to the activation of multiple signaling components, including NF-kB and IRF3, and the subsequent production of pro-inflammatory cytokines (27, 28).

As a non-toxic contaminant stimulus, blank poly(lactide-co-glycolide) microparticles (MP) were employed based on previous evidence of safety and the capacity to trigger endocytic/phagocytic processes (23).

Upon exposure to such stimuli, SC were characterized by addressing the activated mediators and identifying the underlying signalling pathways.

## Materials and Methods

### Primary Cultures of Neonatal Porcine Sertoli Cells

Animal studies were conducted in agreement with the guidelines adopted by the Italian Approved Animal Welfare Assurance (A-3143-01) and European Communities Council Directive of 24 November 1986 (86/609/EEC). The experimental protocols were approved by the University of Perugia. Number 2 large white neonatal pigs (15 to 20 days old) underwent bilateral orchidectomy after general anesthesia with ketamine (Ketavet 100; Intervet, Milan, Italy), at a dose of 40 mg/kg, and dexmedetomidine (Dexdomitor, Orion Corporation, Finland), at a dose of 40 g/kg, and were used as SC donors. Specifically, pure porcine neonatal SC were isolated, characterized and tested for functional competence according to previously established methods (29, 30).

### Microparticle Preparation-Size and Uptake Process

MP were prepared by spray-drying of an acetonitrile polymer solution by using a Buchi Mini spray-dryer B290 (Buchi, Italy). The polymer employed was poly (DL-lactide) (PLA)

R203H (MW 20–30 kDa, Boehringer Ingelheim, Germany). The instrumental parameters were set according to previously published experiments (23). Additionally, particle size analysis was performed by an Accusizer C770 particle counter (PSS, Santa Barbara, CA) equipped with an autodilution system. MP were dispersed in 1% w/v Tween 80 solution, slightly sonicated and immediately analyzed. MP size was expressed as volume mean diameter and population spread as span [Eq. (1)]:

(1) $$span=(d_{90}-d_{10})/d_{50}$$

where d_90_, d_10_ and d_50_ are the diameters ≤90%, 10% and 50% of the population distribution, respectively (23).

Subsequently, the MP were morphologically characterized by Scanning Electron Microscopy (SEM) using a FEG LEO 1525 microscope (LEO Electron Microscopy Inc., NY). The acceleration potential voltage was maintained at 10 keV. Samples were suspended in water and placed onto carbon tape coated aluminum stubs. After complete water evaporation the stubs were sputter coated with chromium prior to imaging by a high resolution sputter (Quorum Technologies, East Essex, UK). Coating was performed at 20 mA for 30 s (23).

MP loading into cells was accomplished by exploiting the innate phagocytic capacity of SC.

MP were properly weighed and dispersed into HAMF12 medium by gentle bath sonication in order to avoid foaming and MP flotation. SC were plated at a density of 0.5–1 × 10^5^ cells/cm^2^ and incubated in standard conditions (37°C, 5% CO2) with MP at a concentration of 30μg/cm^2^ for 5 h according to Giovagnoli et al. (23), obtaining the MP-SC.

### Experimental Design

The experimental groups were assigned as follow:

- Control: untreated SC
- MP-SC
- SC plus LPS (LPS) at concentration of 1μg/ml for 5 h (31).
- MP-SC plus LPS at concentration of 1μg/ml for 5 h.

### Quantitative, Real−Time PCR

Real-time PCR analyses for IDO, PD-L1, TLR-3, TLR-4, MHC-II, ICAM-1, TFGβ1, BDF123, BDF129, BDF3 and pEP2C were conducted as previously described (32) by employing the primers listed in **Table 1**. Briefly, total RNA was extracted, using Trizol reagent (Sigma-Aldrich, Milan, Italy) and quantified by reading the optical density at 260 nm. In particular, 2.5μg of total RNA was subjected to reverse transcription (RT Thermo Scientific, Waltham, MA, USA) in a final volume of 20 μL. Real-time PCR was performed using 25 ng of cDNA prepared by the RT reaction and SYBR Green master mix (Stratagene, Amsterdam, the Netherlands). This procedure was performed in an Mx3000P cycler (Stratagene), using FAM for detection and ROX as reference dye. The mRNA level of each sample was normalized by β-actin mRNA and expressed as fold changes vs the level of the control group.

**Table 1:** Primer sequences for PCR analyses.

Gene	Forward	Reverse	*T_a_*
β-actina	ATGGTGGGTATGGGTCAGAA	CTTCTCCATGTCGTCCCAGT	56°C
IDO	ATGAAGGCGTTTGGGACACC	GAGGAATCCAGCAGCAGAGC	56°C
PDL-1	AAACAATTAGACCTGGCTG	TCTTACCACTCAGGACTTG	56°C
FAS-L	GCAGAAGGAACTGGCAGAAC	TTAAATGGGCCACACTCCTC	56°C
MHCII	GACCAGATGAGGTTATTGG	GGTCCTGTAGTTGTGTCT	56°C
ICAM-1	AGGGAAACCAGACACAAG	ACGACAAGTTAGCCAGTT	56°C
TLR-3	CACTATGCTCGATCTTTCCTAC	CAATTCAGGTACCTCACATTG	56°C
TLR-4	CTTCACTACAGAGACTTCA	ACAATAACCTTCCGACTT	56°C
TGF1β	GCCCTGGACACCAACTATTGC	GCTGCACTTGCAGGAGCGCAC	56°C
TGF3β	GCACTTGCAAAGGGCTC	TTGGCATAGTATTCCGA	56°C
BDF123	GAGTGCGTTGGGAAGATG	TCGGTATGTACTTGGGATGT	56°C
BDF129	TGAAGAGGTCGCCAAGAA	GGATGATGGTGGTGTTGATG	56°C
BDF3	GCCTTGCTCTTCTTGTTC	GCTACCTATCTGTTCCTCTT	56°C
BDF4	GCTACCTATCTGTTCCTCTT	GCATCAAGGTCATTTCTCA	56°C
pEP2C	CAAGTCTCACCTGTTACG	ATCTGCCTTCACTTCTCT	56°C

### Protein Extraction and Western Blot Analysis

Total protein extracts were prepared by lysing cells in 100 μl of radio-immunoprecipitation assay lysis buffer (Santa Cruz Biotechnology Inc., Santa Cruz, CA, USA).

After centrifuging the mixture at 1,000×g (Eppendorf, NY, USA) for 10 min, the supernatant was collected, and total protein content was assayed by the Bradford method (33). Sample aliquots were stored at −20°C for WB analysis. The cell extracts were separated by 4–12% SDS-PAGE, and equal amounts of protein (70 μg protein/lane) were run and blotted on nitrocellulose membranes (BioRad, Hercules, CA, USA). The membranes were incubated overnight in a buffer containing 10 mM TRIS, 0.5 M NaCl, 1% (v/v) Tween 20 (Sigma-Aldrich), rabbit anti-ERK1/2 (Millipore, MA, USA; dilution factor, 1:2,000), mouse anti-phospho- ERK1/2 (Millipore; dilution factor, 1:100), rabbit anti-NF-kB p65 antibody (AbCam, Cambridge, UK; dilution factor, 1:1,000).

Primary antibody binding was then detected by incubating the membranes for an additional 60 min in a buffer containing horseradish peroxidase conjugated anti-rabbit (Sigma-Aldrich; dilution factor, 1:5,000) and/or anti-mouse (Santa Cruz Biotechnology Inc.; dilution factor, 1:5,000) IgG secondary antibodies. The bands were detected by enhanced chemiluminescence.

### Statistical Analysis

Values reported in the figures are the mean ± S.D. of three independent experiments, each one performed in triplicate. Statistical analysis was performed by the paired Student’s t-test using SigmaStat 4.0 software (Systat Software Inc., CA, USA). All tests were performed in triplicate, and statistically significance was assigned to p < 0.05.

## Results

### Sertoli Cell Purification, Characterization, and Function

SC isolated from testes of Large White neonatal pigs were comprised of highly purified tissue (95%) as indicated by the immunostaining for AMH, a specific and unique neonatal SC marker.

The presence of “contaminating” non-SC cells was extremely low (<5%) according to Arato et al. (34).

### Microparticle Dimensional Analysis and Uptake Process

The average size of MP was consistent with possible uptake by SC.

**Figure 1:**
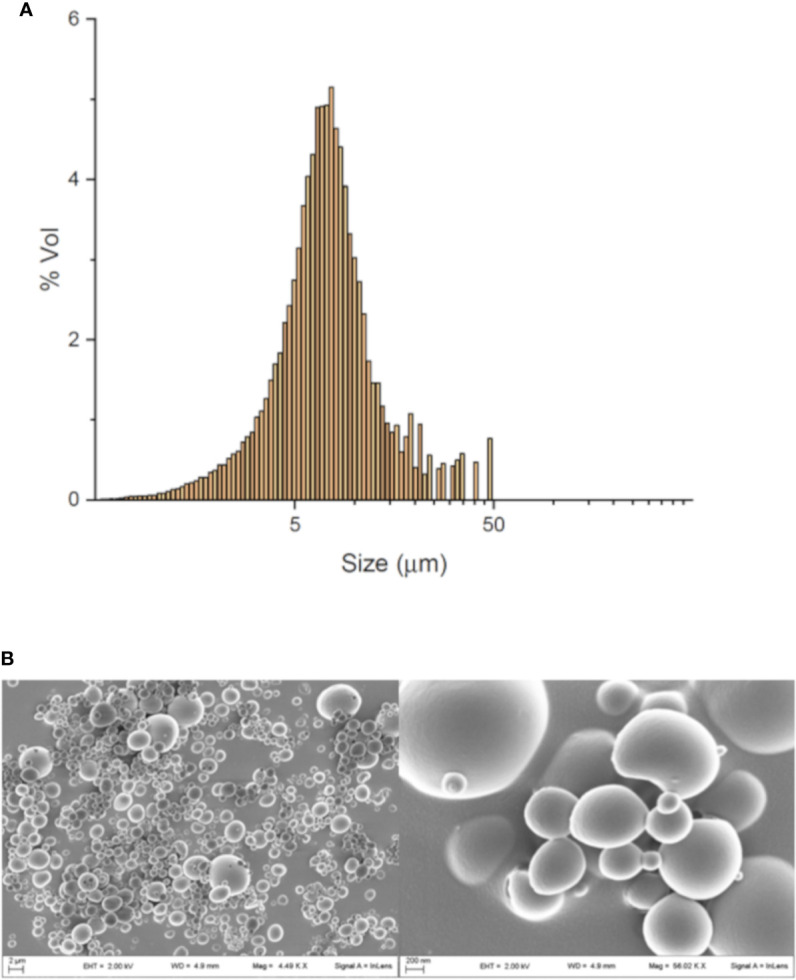
MP characterization and uptake. Dimensional analysis obtained by granulometer **(A)** and SEM image of MP **(B)**.

The average volume mean diameter of MP was 8.8 μm with a span value of 1.2, which indicates a good homogeneity of the particle size distribution as also shown by SEM analysis (**Figure 1**).

### RT-PCR Analysis

As revealed by real-time PCR analyses, a general major increase of gene expression was observed for nearly all the selected mediators mainly when LPS stimulus was employed with MP loading.

**Figure 2:**
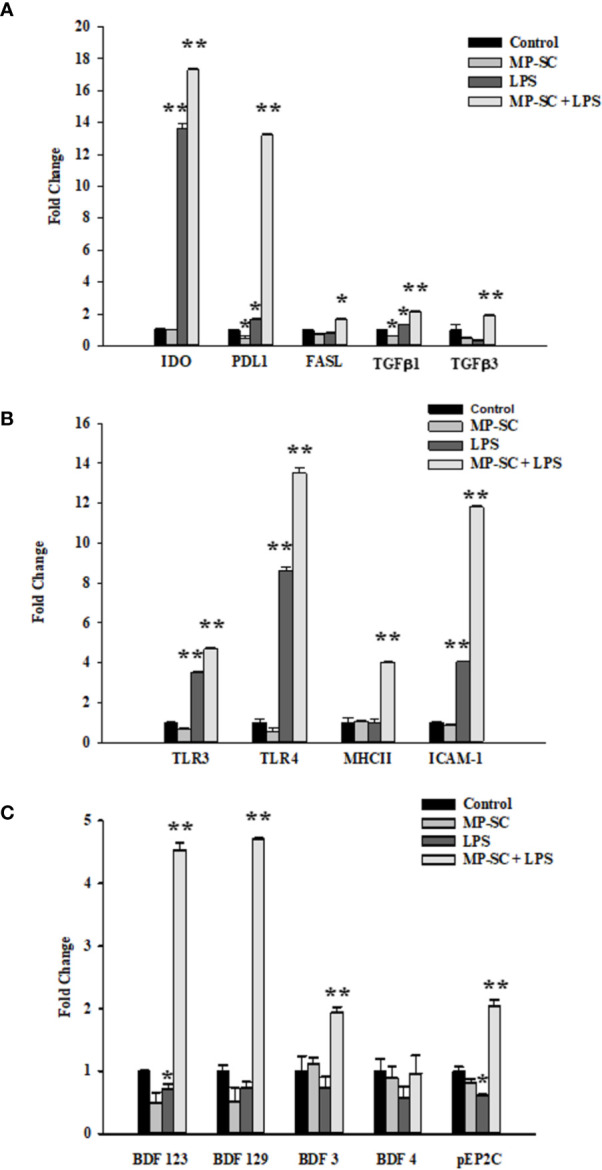
Real-Time PCR. Gene expressions of IDO, PD-L1, FAS-L, TFGβ1, TFGβ3 **(A)** TLR-3, TLR-4, MHC-II, ICAM-1 **(B)** BDF123, BDF129, BDF3, BDF4 and pEP2C **(C)** were evaluated by Real Time PCR. See text for more details. Data represent the mean ± S.E.M. (*p < 0.05 and **p < 0.001 respect to untreated SC) of three independent experiments, each performed in triplicate.

In fact, *IDO* **** gene expression did not changes in MP-SC, while it was significantly higher compared with the control after LPS and MP-SC plus LPS stimulation (**Figure 2A**, p < 0.001). A similar trend was recorded in Programmed Death-Ligand 1 (*PD-L1*)**** expression that increased with LPS and, in particular upon MP-SC plus LPS stimulation, while it resulted even down-regulated in MP-SC (**Figure 2A**, p < 0.05 and p < 0.001). On the other hand, the Fas ligand (*FAS-L* ****) was slightly more expressed only when coupling the MP-SC and LPS treatment (**Figure 2A**, p < 0.05). Likewise, the measurement of the Toll-Like Receptor (*TLR-3*)**** and (*TLR-4*)**** expression confirmed a major effect of LPS and MP-SC plus LPS treatments as compared with the control and no changes in MP-SC (**Figure 2B**, p < 0.001). Moreover, we observed a significant increase in the expression of the Major Histocompatibility Complex (*MHC-II*)**** in MP-SC plus LPS-stimulated SC and not in the other experimental groups. (**Figure 2B**, p < 0.001). The Intercellular Adhesion Molecule 1****(*ICAM-1*) and *TGFβ1* **** expression were upregulated following LPS and MP-SC plus LPS treatments, while only TGFβ1 was down-regulated in MP-SC (**Figures 2A, B**, p < 0.05 and p < 0.001).

The same behavior was observed for BDF123, BDF129, BDF3 and pEP2Cexpression following LPS and MP-SC plus LPS treatment with no changes in MP-SC. The only exception was BDF123 and pEP2C that were down-regulated by LPS treatment (**Figure 2C**, p < 0.05 and p < 0.001).

### Western Blot Analysis

**Figure 3:**
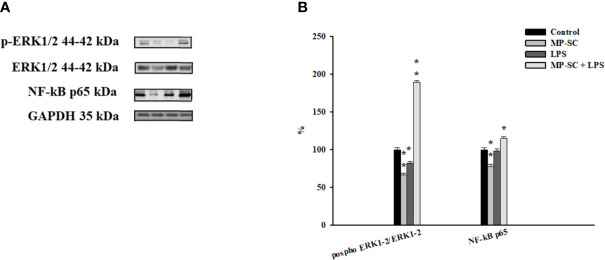
WB analysis. Immunoblots of phospho-ERK1-2/ERK1-2 and NF-kB p65 **(A)**. Densitometric analysis of the protein bands of phospho- ERK1-2/ERK1-2 and NF-kB p65 **(B)**. Data represent the mean ± S.E.M. (*p < 0.05 and **p < 0.001, respect to untreated SC) of three independent experiments, each performed in triplicate.

The WB recorded showed a general increase of the phosphorylation ratio of ERK1-2 compared to the control by MP plus LPS treatment, while we observed a significant reduction after MP-SC and LPS treatment (**Figures 3A, B**, p < 0.05 and p < 0.001).

On the other hand, the Nf-kB p65 level was significantly up-regulated only for MP-SC plus LPS treatment, with a statistically significant reduction after MP-SC treatment and not changes after LPS stimulus compared to the control (**Figures 3A, B**, p < 0.05 and p < 0.001).

## Discussion

In the present study, we focused on the effect of the exposure of SC to MP and LPS as models of non-toxic and infection-released stimuli, respectively, to understand changes in SC signatures due to the activation of selected mediators and signalling pathways.

In particular, our experimental design consisted in a first step in which SC were loaded with empty MP mimicking what reported by Giovagnoli et al. (23). that could be followed or not by a second step of stimulation with LPS to create a useful high mammalian *in vitro* model to assess the cell responses at an early stage of infection by i.e. Gram-negative bacteria or mycobacteria (31). In fact, LPS is a standard agent employed in *in vivo* and *in vitro* protocols acting as a potent stimulator of the innate immune response (28). This setup was intended to simulate cell activation against pro-inflammatory and immunostimulatory agents (23).

MP loading was easily achieved by exploiting the natural scavenging role of SC in the testis. In fact, it is well known that, under physiologic conditions, SC internalize residual cytoplasmic bodies as an important function to maintain the homeostasis of the testis (35, 36).

In response to LPS stimulation of MP-SC, we observed up-regulation of gene expression for important markers involved in the innate immune response such as IDO, PD-L1, FAS-L, TLR-3, TLR-4, MHC-II, ICAM-1, TFGβ1, BDF123, BDF129, BDF3 and pEP2C.

*IDO* interferes with immunity as a result of its involvement in the kynurenine pathway (the O_2_-dependent oxidation of L-tryptophan) and it increases the production of Treg, involved in the surveillance of self-tolerance to auto-antigens, thus preventing autoimmunity (37).

Fallarino et al., 2009, for the first time, demonstrated IDO’s expression in SC (9). Herein, the up-regulation of IDO gene expression, after subsequent stimulation with LPS, would explain a possible role of SC in immuno-regulation, within a TGFβ mediated IDO-dependent mechanism as demonstrated by the further up-regulation of *TGFβ1* **** gene expressions (9).

*PD-L1* ****, also known as CD274 or B7-H1, is a fundamental trans-membrane protein involved in the repression of the immune response during pregnancy, allograft, autoimmune diseases and other diseases such as hepatitis (38). The expression of B7-H1 contributes to the inhibition of immune responses by negatively interfering with CD8+ T cell proliferation, while inducing the expression of MHC class II, which mediates the increase of Tregs (39), as also demonstrated by the observed up regulation of MHC-II and ICAM1 gene expression, confirming previous published reports (39). In fact, *MHC*-*II* is a well-known trans-membrane protein and its main function is to present processed antigens, which are derived primarily from exogenous sources, to CD4(+) T-lymphocytes, playing an essential role in innate as well as acquired immunity (40). *ICAM-1*, also known as CD54, is a cell surface glycoprotein expressed on a wide variety of cell types, with distinct patterns of gene regulation and effector functions. ICAM-1 is expressed constitutively at low levels in endothelial cells and some lymphocytes and monocytes and its expression can be significantly increased, in the presence of cytokines, even in nonvascular cells such as SC (5, 31).

In our model, the increased expression of these mediators seems to confirm the data reported by Dal Secco et al, showing how SC could function as nonprofessional tolerogenic antigen-presenting cells by inducing enrichment in regulatory T cells (Tregs) in a mixed T lymphocyte population (39).

All these data were further corroborated by upregulation of *FAS-L* gene expression that is a trans-membrane protein belonging to the TNF (Tumor necrosis factor) family regulating the immune response by formation of the immunological synapse (41).

Additionally, our experimental setup confirmed the involvement of TLR-4 in LPS stimulation of MP-SC, where *TLR-4* is a transmembrane protein capable of recognizing specific pathogen molecules such as LPS of mycobacteria wall and Gram-negative bacteria. Specifically, LPS exerts its effects exclusively through the TLR-4 receptor complex (28) leading to activation of the intracellular signaling pathway of NFkB, responsible for the activation of the innate immune system, as confirmed by our WB data analysis where we observed the up-regulation of *NF-kB p65*, an important effector of canonical activation of the NF-kB pathway (5, 28).

Furthermore, we observed an increased gene expression of *TLR-3*, another member of the TLR family that plays a fundamental role in the recognition of non-self (DAMP/PAMP) molecules, by activating the innate immune response (5). These observations confirmed the data of Qin et al. showing how TLR3 upregulation contributed on the modulation of inflammatory cytokine generation during orchitis in testicular cells (42).

In the present study, we focused, additionally, on analyzing the expression of β-defensins (BDF) by MP-SC after LPS treatment.

*β-defensins*, a major group of mammalian antimicrobial peptides, are expressed in SC and represent one of the earliest mediators of the host’s defense in human and animals (19). The expression of β-defensins in the testis and different regions of the epididymis provides an innate defense immune mechanism in the male reproductive tract (43). In European wild boar naturally infected with *Mycobacterium bovis*, Galindo et al. (40) observed an overexpression of BDF129 suggesting its protective role against mycobacteria and confirming the data of Riva-Santiago et al. (44). who observed the overexpression of BDF3 and BDF4 mRNA, in a mouse model of mycobacterial infection, thus hypothesizing their role in the control of mycobacterial growth.

In our study, we observed upregulation of *BDF123, BDF129, BDF3* and *pEP2C*, confirming their role in the innate defense immune mechanisms in the male reproductive tract (44).

Finally, our WB data, demonstrated un up-regulation of phosphorylation ratio of ERK 1-2.

The increase of ERK, member of the mitogen-activated protein kinases (MAPK) pathway, suggest their involvement in LPS stimulation.

There are several studies showing that MAPK play an important role in numerous male reproductive processes, including BTB dynamics, germ cell-cycle progression and differentiation, and germ cell apoptosis in the seminiferous epithelium (45, 46).

In particular, previous studies showed that BTB dysfunction is closely associated with the activation of the MAPK and NFκB pathways in some infections (5, 47, 48).

In conclusion, our data confirmed that SC are able to uptake MP, as previously described (23), and may be subsequently activated by LPS stimulation (31), suggesting that this experimental procedure seems to be the best strategy to activate SC against a microbial agent (synergistic effect).

Moreover, the ability of SC to internalize MP could be exploited by using them as carriers to deliver drugs to the target area, with no systemic side effects.

The present work, by pinpointing the exponential increase of pro-inflammatory pathways in MP-SC, after LPS stimulation, suggests a hypothetical “role” of SC, additional to that of cells of the blood-testicular barrier, as nonprofessional tolerogenic antigen-presenting cells.

All these data strengthen the concept that SC can be considered a complex “micro-laboratory” secreting a cocktail of immunomodulatory factors.

These findings warrant further investigation to understand how the SC respond to infections by activating several pro-inflammatory pathways.

A prospective impact of the present study is the possibility to transform SC into potentially valuable therapeutic tools by exploring their native and induced competences. Unraveling the effect of external stimuli on SC competence is crucial to depict the underlying response mechanisms that may be useful to tackle diseases with inflammatory or immunological signatures even of infective origin. The dual capacity of SC to function as carries as well as immunomodulatory/antibacterial agents could be exploited to develop novel cell-based treatments for pathologies otherwise difficult to treat. Such an ambitious goal cannot be sought without knowing how SC manipulation alters their natural features and without a full disclosure of the clinical relevance of such observations.

This study represents a first attempt to comprehend the intricate pattern of SC stimulation that naturally occurs when cells are exposed to non-physiological conditions or are manipulated in laboratory settings. As such, this study shows several limitations as it lacks of insightful quantification of cell signaling pathways and secretome analysis. In this regard, future studies are desirable implying the application of advanced molecular techniques, including proteomics, single-cell RNA analysis and macroarray investigations to identify the possible involvement of secondary factors and pathways to broaden the picture on induced sertolian functions.

## Data Availability Statement

The raw data supporting the conclusions of this article will be made available by the authors, without undue reservation.

## Ethics Statement

The animal study was reviewed and approved by Italian Approved Animal Welfare Assurance (A-3143-01).

## Author Contributions

All authors had critically revised and approved the final version of the manuscript. IA and DM designed and drafted the manuscript. The experimental procedures and data analysis were performed by CB, CL, SB, SC, PM, MC, and TB, SG, GG, and RC gave experimental guidance. FM and GL supervised and revised the manuscript. All authors contributed to the article and approved the submitted version.

## Conflict of Interest

The authors declare that the research was conducted in the absence of any commercial or financial relationships that could be construed as a potential conflict of interest.
